# Effect of Neurokinin-1 Receptor Antagonists on Experimental Postoperative Adhesion: A Systematic Review and Meta-Analysis with Trial Sequential Analysis of Preclinical Studies

**DOI:** 10.3390/medicina61111933

**Published:** 2025-10-28

**Authors:** Hyeon Joung Hwang, Geun Joo Choi, Yoo Shin Choi, Beom Gyu Kim, Hyun Kang

**Affiliations:** 1 Department of Anesthesiology and Pain Medicine, College of Medicine, Chung-Ang University, 84 Heukseok-ro, Dongjak-gu, Seoul 06974, Republic of Korea; shine1@cau.ac.kr (H.J.H.); pistis23@cau.ac.kr (G.J.C.); 2 Department of Surgery, College of Medicine, Chung-Ang University, 84 Heukseok-ro, Dongjak-gu, Seoul 06974, Republic of Korea; choiys@cau.ac.kr (Y.S.C.); kimbg0526@cau.ac.kr (B.G.K.)

**Keywords:** Neurokinin-1 receptor antagonist, substance P, tissue adhesions, meta-analysis as topic, systematic review, trial sequential analysis

## Abstract

*Background and Objectives*: Postoperative adhesions are a major surgical concern. Preclinical studies of neurokinin-1 receptor antagonists (NK-1RAs) have reported inconsistent findings. We conducted a systematic review and meta-analysis with trial sequential analysis (TSA) to evaluate whether NK-1RAs prevent postoperative adhesions. *Materials and Methods*: We searched MEDLINE (via PubMed), EMBASE (via Ovid), Web of Science, and Google Scholar for animal studies assessing NK-1RAs applied to the surgical site. The primary outcome was macroscopic adhesion score; secondary outcomes were tissue plasminogen activator (t-PA) and plasminogen activator inhibitor-1 (PAI-1) mRNA expression in peritoneal tissue and t-PA activity in peritoneal tissue. *Results*: Nine studies including 331 animals (162 NK-1RA; 169 control) met the criteria. Macroscopic adhesion scores were significantly lower with NK-1RAs than control (standardized mean difference [SMD] 1.474; 95% confidence interval [CI] 1.030 to 1.918; P_chi_^2^ = 0.001; I^2^ = 63.8%; τ = 0.619; 95% predictive interval [PrI] 0.133 to 2.815). TSA showed the cumulative Z-curve crossed both the conventional and trial sequential monitoring boundaries for the macroscopic adhesion score. t-PA activity was higher with NK-1RAs (SMD −1.219; 95% CI −2.379 to −0.058; P_chi_^2^ < 0.001; I^2^ = 77.66%; τ = 1.453; PrI −4.655 to 2.217). There was no evidence of a difference in t-PA or PAI-1 mRNA expression. *Conclusions*: NK-1RAs reduced postoperative adhesions by macroscopic scoring and increased t-PA activity in preclinical models.

## 1. Introduction

Postoperative adhesion formation is part of normal tissue repair after surgery [1]. However, adhesions develop in an estimated 60–93% of patients undergoing abdominal operations and are linked to small bowel obstruction, female infertility, technical difficulty during reoperations, chronic abdominal or pelvic pain, and, rarely, paraplegia [2,3]. These sequelae often necessitate additional surgical interventions, increase readmissions, prolong hospitalization, and substantially raise healthcare costs [4].

Multiple preventive strategies have been explored over recent decades. These include gentle surgical technique, frequent intraoperative irrigation [5], and physical barriers applied to injured surfaces [6] in the form of films, solutions, and gels [7]. Chemical approaches have also been tested, such as statins [8], nonsteroidal anti-inflammatory drugs, heparin [9], fibrinolytic agents [10], thrombin-activated fibrinolysis inhibitors [11], methylene blue [12], local anesthetics [13], antibiotics [14], and combinations of mechanical and chemical barriers.

In clinical practice, the most common strategy is placement of a physical barrier around the surgical site. Although effective locally, barriers are not universally reliable and do not protect remote peritoneal surfaces, leaving patients at risk for adhesions elsewhere. This limitation supports evaluating chemical strategies that act throughout the peritoneal cavity.

Neurokinin-1 receptor antagonists (NK-1RAs, “-pitants”) are established antidepressant, anxiolytic, and antiemetic agents. Evidence suggests that the neurokinin-1 receptor (NK-1R) pathway, activated by substance P, contributes to adhesion formation via inflammation, oxidative stress, and impaired fibrinolysis. By blocking substance P binding to NK-1R, NK-1RAs may restore peritoneal fibrinolytic balance and attenuate adhesion-promoting cascades. Preclinical studies of NK-1RAs have reported mixed results, and no prior systematic review or meta-analysis has synthesized their efficacy for adhesion prevention.

This study aimed to conduct a systematic review and meta-analysis with trial sequential analysis (TSA) to critically evaluate preclinical evidence. We focused on NK-1RAs administered at the surgical site and their effect on postoperative adhesions.

## 2. Materials and Methods

### 2.1. Protocol and Registration

We developed this protocol in accordance with preferred reporting items for systematic review and meta-analysis protocols (PRISMA-P) and prospectively registered it in PROSPERO (CRD42021211602; www.crd.york.ac.uk/Prospero) on 22 August 2025.

This systematic review and meta-analysis with TSA on NK-1RAs for experimental postoperative adhesion followed Cochrane Collaboration methods [15] and adhered to the PRISMA 2020 reporting guideline [16]. The complete PRISMA 2020 checklist is provided as Supplementary Materials.

### 2.2. Eligibility Criteria

We prespecified inclusion and exclusion criteria. We included animal studies comparing NK-1RAs applied to the surgical site with a control for prevention of postoperative adhesions.

PICO-SD was defined as follows:

Animals/Population (P): all animals undergoing surgery.

Intervention (I): NK-1RAs applied to the surgical site.

Comparisons (C): no pharmacological exposure (e.g., no NK-1RA, normal saline, or distilled water) or use of the same materials as the intervention group without NK-1RA.

Outcome (O): primary outcome, macroscopic adhesion score; secondary outcomes, tissue plasminogen activator(t-PA) and plasminogen activator inhibitor(PAI)-1 mRNA expression in peritoneal tissue, t-PA activity in peritoneal tissue, and anastomotic bursting pressure.

Study design (SD): controlled studies with separate treatment groups.

Exclusion criteria were as follows: (1) non-animal studies (ex vivo, in vitro, or human); (2) interventions other than NK-1RAs; (3) no relevant outcomes; (4) no control group; and (5) reviews, case reports, case series, letters, or commentaries.

### 2.3. Literature Search

Two investigators independently searched MEDLINE (via PubMed), EMBASE (via Ovid), Web of Science, and Google Scholar in August 2025. The strategy combined free text, Medical Subject Headings, and EMTREE terms (Appendix A.1—Search terms). Records were imported into EndNote 9.3 (Thomson Reuters, Philadelphia, PA, USA) and duplicates removed. We screened reference lists of included articles and relevant reviews. No language or date limits were imposed. When needed, we planned to collaborate with institutional experts for translation.

### 2.4. Study Selection

Two investigators independently screened titles and abstracts. Reports deemed potentially eligible by either reviewer proceeded to full-text assessment. We also reviewed conference proceedings. To minimize duplication, we compared papers from the same authors, institutions, or countries. Studies meeting inclusion criteria were evaluated independently by two investigators; disagreements were resolved by discussion or, if needed, by a third investigator.

### 2.5. Data Extraction

Using a standardized extraction form, two investigators independently extracted data with subsequent cross-validation. Disagreements were resolved by re-review and, if necessary, arbitration by a third investigator.

We classified any NK-1RA administered at the surgical site—regardless of agent, dose, or method—as the NK-1RA group. Controls included no pharmacologic exposure (e.g., no NK-1RA, normal saline, or distilled water) or identical materials without NK-1RA. If a study included multiple eligible NK-1RA arms contributing to the overall effect, the data from the shared control group were proportionally divided among the intervention arms to avoid unit-of-analysis errors. In studies with four or more groups where anti-adhesion materials (e.g., films, membranes, or sponges) were applied equally or not applied across NK-1RA and control groups, data were extracted as separate sub-studies stratified by material use.

Extracted items included the following: (1) title; (2) first author; (3) journal; (4) year; (5) animal species; (6) surgical model; (7) control intervention; (8) experimental intervention (type and dose of NK-1RA); (9) definition of macroscopic adhesion score; (10) macroscopic adhesion score; (11) t-PA mRNA expression; (12) PAI-1 mRNA expression; (13) t-PA activity; (14) anastomotic bursting pressure; and (15) methodological quality.

When data were missing or incomplete, we contacted study authors. For numerical data presented only in figures, we used Plot Digitizer (version 2.6.8; http://plotdigitizer.sourceforge.net (accessed on 1 September 2025)) to extract values.

### 2.6. Methodological Quality and Publication

We assessed five domains: (1) random allocation; (2) husbandry conditions (light/dark cycle, temperature, access to water, environmental enrichment); (3) compliance with animal welfare regulations; (4) potential conflicts of interest; and (5) peer-reviewed publication status. Two investigators independently scored each study from 0 to 5; disagreements were resolved by a third investigator.

In addition, we further evaluated the methodological quality using SYRCLE’s Risk of Bias (RoB) tool [17], which is specifically designed for animal intervention studies.

When a commercially supplied compound was used and no information regarding funding or conflicts of interest was reported, the corresponding items were downgraded (from 1 to 0) or rated as unclear risk to reflect limited transparency rather than definite bias

### 2.7. Statistical Analyses

We summarized study characteristics in ad hoc tables. Two investigators independently entered data for analysis. For the overall analysis, standardized mean differences (SMDs) with 95% confidence intervals were used because the included studies employed different scoring systems for adhesion severity and different units for biochemical outcomes. In subgroup analyses stratified by animal species, where identical scoring systems were used (percent in rats and 5-point ordinal scale in mice), mean differences (MDs) were calculated.

Data from animal studies were pooled under the assumption of comparable pharmacologic mechanisms of NK-1 receptor antagonism across species, dosages, and surgical models. Random-effects models were used to account for potential biological and methodological heterogeneity.

We assessed heterogeneity with Cochran’s Q, Higgins’ I^2^, τ (DerSimonian–Laird), and prediction intervals (PrI) [18]. Heterogeneity was considered substantial if Cochran’s Q yielded *p* < 0.10 or I^2^ > 50% [19]. When outcomes show heterogeneity and the number of combined comparison was fewer than 10, we used t-statistics (Hartung–Knapp–Sidik–Jonkman method) instead of Z-tests to reduce type I error [20].

We conducted subgroup analyses by animal species and surgical model (ischemic button or laparoscopic lysis after intraperitoneal ischemic buttons vs. laparotomy with cecal cautery). Sensitivity analyses excluded one study at a time. When studies reported medians (range, P_25_–P_75_), medians (ranges), or means (standard errors), we derived means and standard deviations(SDs) using established methods [21].

Publication bias was assessed visually with funnel plots and, considering small-study effects, with Egger’s linear regression test. Evidence of bias was considered when funnel plots were asymmetric or Egger’s test yielded *p* < 0.10. When fewer than 10 studies were available, we did not assess publication bias.

### 2.8. Trial Sequential Analysis

We performed TSA for the macroscopic adhesion score to estimate the required information size (RIS) and determine whether evidence was conclusive. Using a random-effects model, we constructed the cumulative Z-curve and maintained a 5% overall type I error. If the cumulative Z-curve crossed the monitoring boundary or entered the futility area, we considered the evidence sufficient; if it did not cross any boundary and the RIS was not reached, further studies were needed [22].

For the macroscopic adhesion score, we used the observed SD, a mean difference of observed SD/3, alpha of 5%, beta of 10%, and observed diversity from the meta-analysis. These assumptions were chosen to reflect a moderate expected effect and to balance statistical sensitivity with the small and heterogeneous preclinical dataset.

Traditional meta-analysis and meta-regression were performed in Comprehensive Meta-Analysis (version 2.0; Biostat, Englewood, NJ, USA). TSA was performed with TSA software 0.9.5.10 (Beta Copenhagen Trial Unit, Centre for Clinical Intervention Research, Copenhagen, Denmark).

### 2.9. Certainty of Evidence

The certainty of evidence for each outcome was assessed according to the framework proposed by Hooijmans et al. [23], which adapts GRADE domains to preclinical animal studies. Certainty was qualitatively rated as high, moderate, low, or very low based on risk of bias, inconsistency, indirectness, imprecision, and publication bias.

## 3. Results

### 3.1. Study Selection

**Figure 1:**
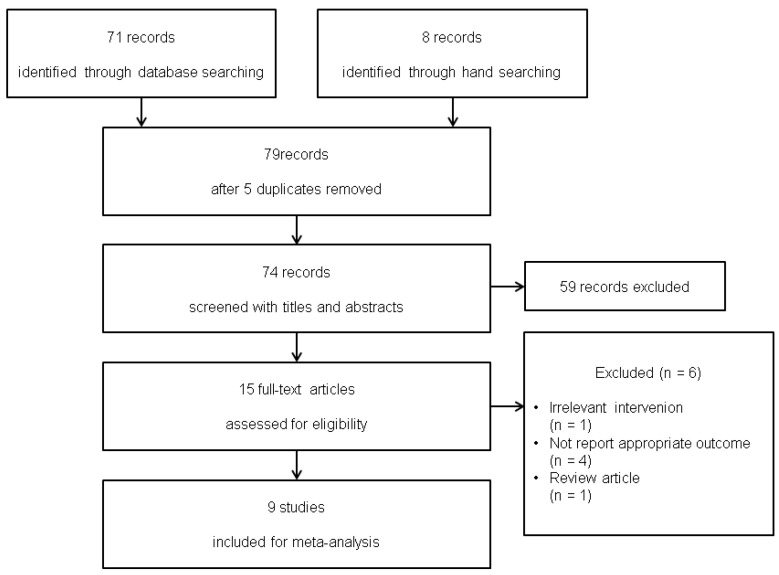
Flow diagram of study selection.

Database searches identified 122 records, with an additional eight from manual searching. After removing duplicates (n = 22), 108 records remained; 93 were excluded at title/abstract screening as irrelevant. Conference proceedings were considered but excluded for reasons listed in Appendix B.1—Conference proceedings excluded. Inter-reviewer agreement at this stage was κ = 0.813. We assessed 15 full-text articles and excluded six, with reasons detailed in Appendix B.2.—Articles excluded at full-text review. Agreement for included articles was κ = 1.000. Nine studies (331 animals; 169 control; 162 NK-1RA) were included (Figure 1).

Study characteristics are summarized in Table 1. Surgical models included intraperitoneal ischemic buttons [24,25,26,27,28,29], laparoscopic lysis of adhesions after intraperitoneal ischemic buttons [30], and laparotomy with cecal cautery [31,32]. Wistar rats [24,25,26,27,28,29,30] and mice [31,32] were used. NK-1RA agents were CJ-12,255 [24,25,26,28,29,30,31,32] and aprepitant [27]. Controls included saline [24,25,28,29,31,32], sterile water [26,30], and dimethyl sulfoxide (DMSO) [27].

### 3.2. Macroscopic Adhesion Score

Nine studies [24,25,26,27,28,29,30,31,32] reported macroscopic adhesion scores. Seven [24,25,26,27,28,29,30] used percent adhesion scores based on the proportion of ischemic buttons with adhesions in rats, and two [31,32] used the 5-point scale of Kennedy et al. [33] in mice (Table 2).

**Figure 2:**
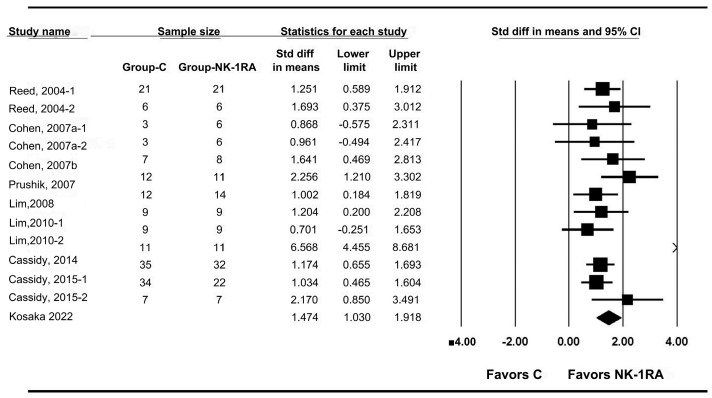
Effect of neurokinin-1 receptor antagonist versus control on macroscopic adhesion score. Each trial is shown as a filled square proportional to sample size, with the 95% confidence interval (CI) depicted as a horizontal line; the diamond indicates the pooled effect and its uncertainty. C, control; NK-1RA, neurokinin-1 receptor antagonist [24,25,26,27,28,29,30,31,32].

Macroscopic adhesion scores were significantly lower in the NK-1RA group than control (SMD 1.474; 95% CI 1.030 to 1.918) with substantial heterogeneity (P_chi_^2^ = 0.001; I^2^ = 63.8%; τ = 0.619; PrI 0.133 to 2.815) (Figure 2).

**Figure 3:**
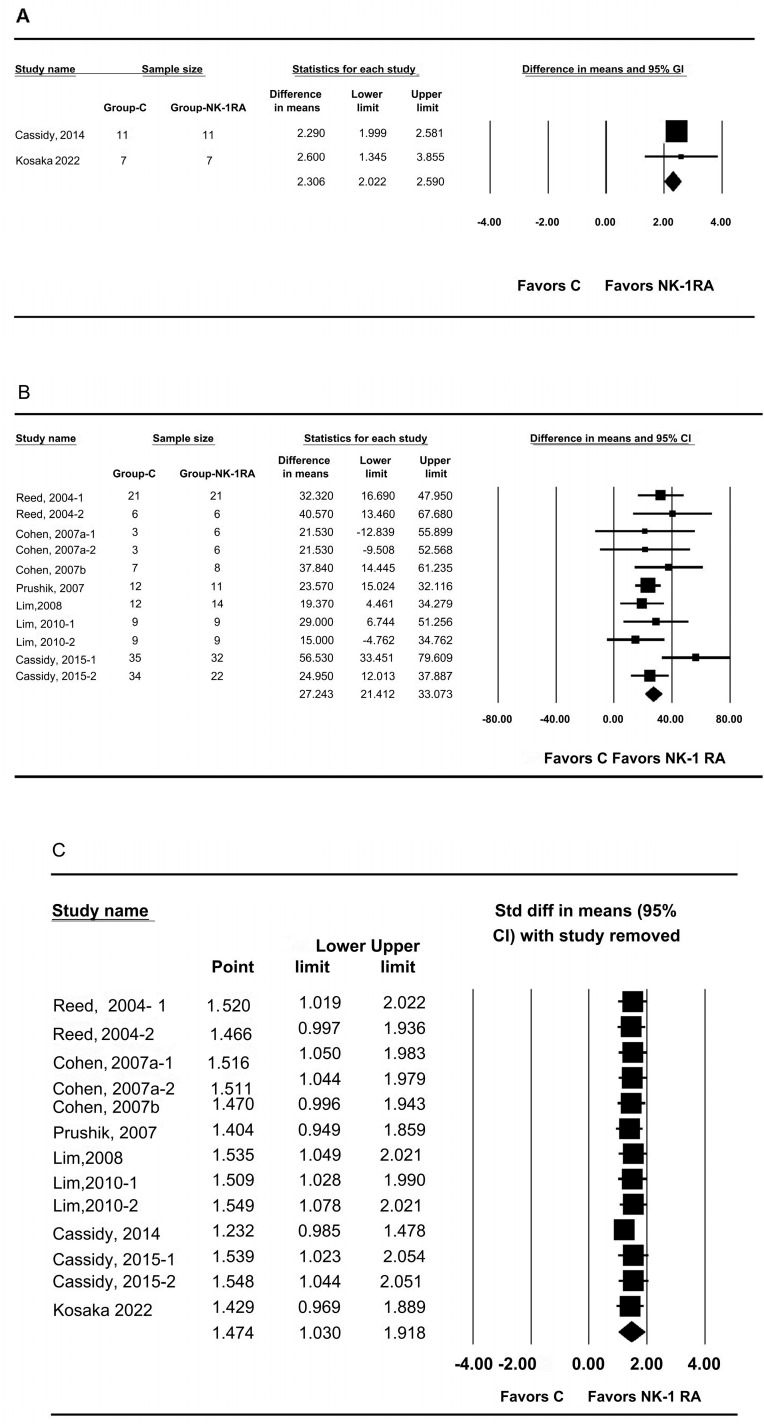
Subgroup and sensitivity analyses of the effect of neurokinin-1 receptor antagonist versus control on macroscopic adhesion score. Panels: (**A**) mouse; (**B**) rat; (**C**) leave-one-out sensitivity analysis. Each trial is shown as a filled square proportional to sample size, with the 95% confidence interval (CI) depicted as a horizontal line; the diamond indicates the pooled effect and its uncertainty. The overall pooled estimate indicated a significantly lower macroscopic adhesion score in the neurokinin-1 receptor antagonist group than in the control group. C, control; NK-1RA, neurokinin-1 receptor antagonist [24,25,26,27,28,29,30,31,32].

Subgroup analyses showed consistent effects with decreased heterogeneity: mouse models (5-point scale) had an MD of 2.306 (95% CI 2.022 to 2.590; P_chi_^2^ = 0.637; I^2^ = 0.0%; τ = 0.0) (Figure 3A), and rat models (percent adhesion score) had an MD of 27.243 (95% CI 21.412 to 33.073; P_chi_^2^ = 0.293; I^2^ = 15.86%; τ = 3.873; PrI 18.613 to 35.872) (Figure 3B). Leave-one-out sensitivity analysis did not change statistical significance (Figure 3C)

**Figure 4:**
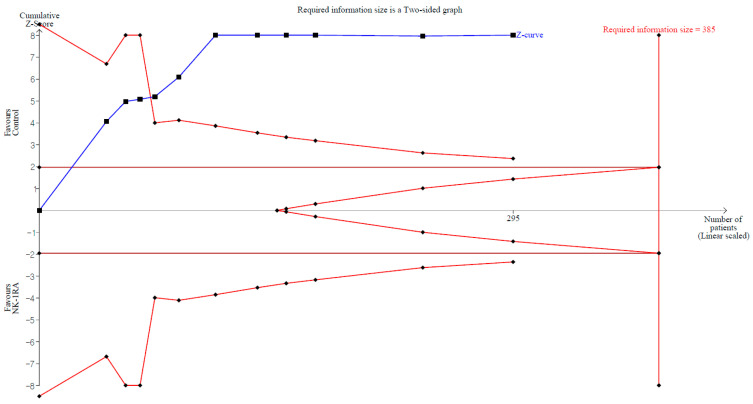
Trial sequential analysis of the effect of neurokinin-1 receptor antagonist versus control on macroscopic adhesion score. The uppermost and lowermost solid red curves denote the trial sequential monitoring boundaries for benefit and harm, respectively. The horizontal dotted red line marks the conventional significance threshold. Triangular red lines at the right indicate the futility boundaries. The vertical solid red line indicates the required information size. The solid blue line is the cumulative z-curve. Numbers on the *x*-axis indicate the required information size.

TSA indicated that 76.6% of the RIS was accrued (295 of 385 animals), and the cumulative Z-curve crossed both the conventional and trial sequential monitoring boundaries (Figure 4).

### 3.3. t-PA and PAI-1 mRNA Expression in Peritoneal Tissue

**Figure 5:**
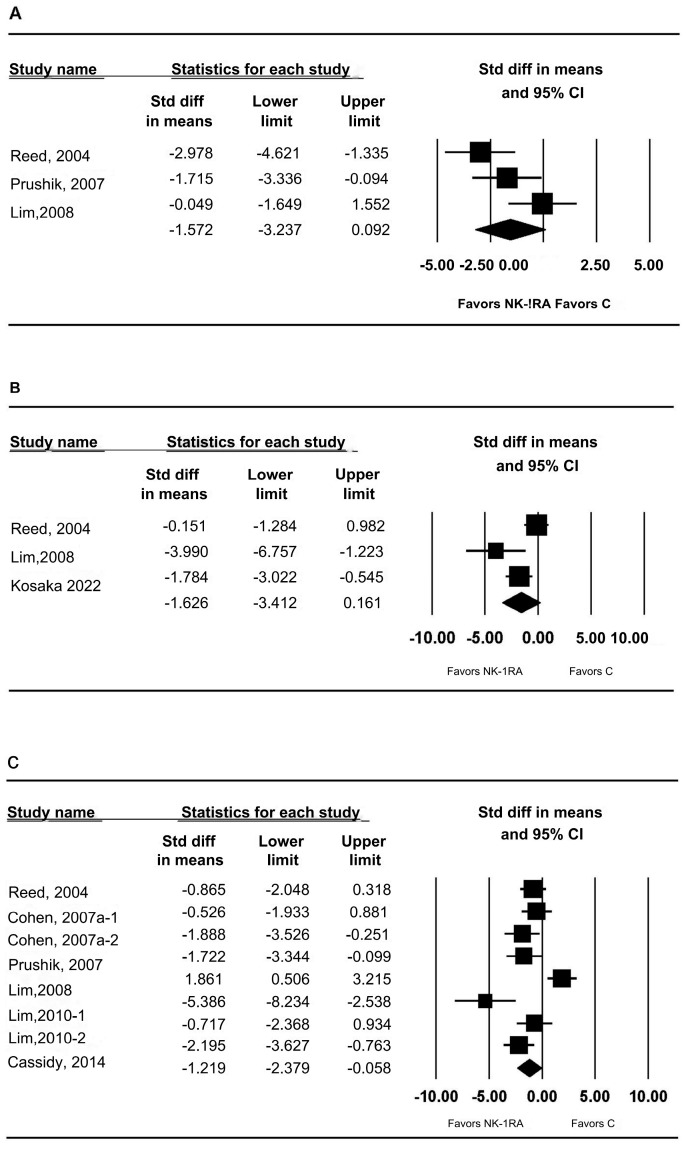
Effect of neurokinin-1 receptor antagonist versus control on (**A**) tPA mRNA expression in peritoneal tissue, (**B**) PAI-1 mRNA expression in peritoneal tissue, and (**C**) tPA activity in peritoneal tissue. Each trial is shown as a filled square proportional to sample size, with the 95% confidence interval (CI) depicted as a horizontal line; the diamond indicates the pooled effect and its uncertainty. C, control; NK-1RA, neurokinin-1 receptor antagonist [24,25,27,28,30,31,32].

Three studies each reported t-PA mRNA [24,27,30] and PAI-1 mRNA [24,27,32]. No statistically significant differences were observed: t-PA mRNA (SMD −1.572; 95% CI −3.237 to 0.092; P_chi_^2^ = 0.042; I^2^ = 65.36%; τ = 1.216; PrI −6.805 to 3.661) (Figure 5A) and PAI-1 mRNA (SMD −1.626; 95% CI −3.412 to 0.161; P_chi_^2^ = 0.018; I^2^ = 75.12%; τ = 1.216; PrI −7.345 to 4.093) (Figure 5B).

### 3.4. t-PA Activity in Peritoneal Tissue

Six studies [24,25,27,28,30,31] measured t-PA activity. NK-1RAs increased t-PA activity versus control (SMD −1.219; 95% CI −2.379 to −0.058; P_chi_^2^ < 0.001; I^2^ = 77.66%; τ = 1.453; PrI −4.655 to 2.217) (Figure 5C).

### 3.5. Anastomotic Bursting Pressure

One study measured anastomotic bursting pressure [27]. There was no evidence of a difference between control and NK-1RA groups (228 ± 27 mm Hg vs. 248 ± 44 mm Hg).

### 3.6. Publication Bias

**Figure 6:**
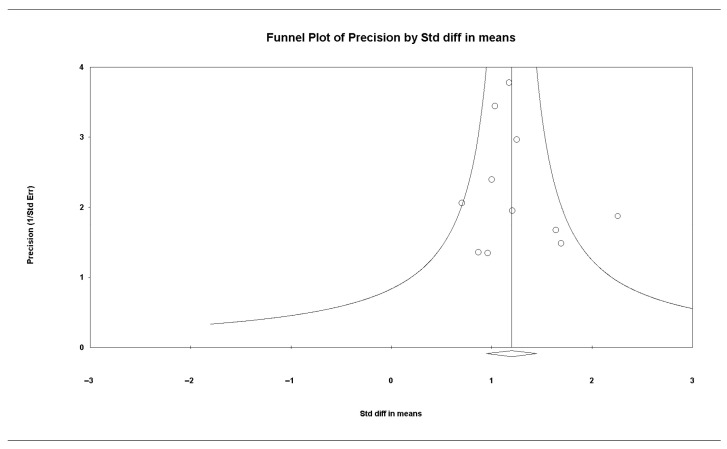
Funnel plot of the effect of neurokinin-1 receptor antagonist versus control on macroscopic adhesion score.

Funnel plots were symmetric for macroscopic adhesion scores, and Egger’s regression showed no evidence of publication bias (intercept, 0.565; 95% CI, −1.141 to 2.271; *p* = 0.473). (Figure 6).

### 3.7. Methodological Quality

Table 3 and Table 4 summarize methodological quality assessed using 5-point scale and SYRCLE’s Risk of Bias (RoB) tool for each study. Inter-rater agreement for methodological quality and risk-of-bias assessments was calculated using Cohen’s kappa statistics (κ = 0.784 for the 5-point scale and κ = 0.844 for the SYRCLE’s RoB tool).

Methodological quality scores assessed using 5-point scale ranged from 3 to 4. Three studies did not report random allocation [27,29,32]. With the exception of Lim et al. [27], which used aprepitant as the NK-1RA, all studies used CJ-12,255 supplied by Pfizer, Inc. (Groton, CT). None of the included studies explicitly declared the absence of conflicts of interest; therefore, potential conflicts of interest were considered and the respective items were downgraded.

Table 4 presents the risk-of-bias assessment performed using SYRCLE’s Risk of Bias (RoB) tool. Across studies, allocation sequence generation, allocation concealment, and random housing or blinding of investigators during outcome assessment were generally not described. Blinding of outcome assessors was not reported in three studies [27,29,30]. Similarly to the methodological quality assessment, all studies except Lim et al. [27] used CJ-12,255 supplied by Pfizer, and none reported conflict-of-interest statements; hence, these domains were judged as having unclear risk.

### 3.8. Certainty of Evidence

The certainty of evidence, assessed using a preclinical GRADE-based framework, ranged from low to very low across outcomes (Table 5).

Macroscopic adhesion and t-PA activity were rated low due to study limitations and heterogeneity, whereas t-PA and PAI-1 mRNA expression were rated very low owing to additional imprecision and inconsistency. Anastomotic bursting pressure was rated low, mainly due to limited precision.

## 4. Discussion

This systematic review and meta-analysis with trial sequential analysis found that NK-1R antagonism reduces postoperative adhesion formation, as shown by macroscopic adhesion scores and t-PA activity in peritoneal tissue. TSA and the predictive interval indicated that the macroscopic adhesion score difference was conclusive. There was no evidence of differences in t-PA or PAI-1 mRNA expression in peritoneal tissue.

Postoperative adhesions are a common, often underrecognized surgical complication that arises from the normal wound-healing response [1]. Although initial fibrinous tissue formation is protective, subsequent dense fibrous bands can have substantial health consequences. Clinically, adhesions are a leading cause of small bowel obstruction, a condition associated with high morbidity and, in severe cases, mortality. They also contribute to chronic abdominal or pelvic pain, infertility in women due to tubal obstruction, and increased risk of iatrogenic injury during repeat surgical procedures [34]. As life expectancy rises, more individuals undergo primary and repeat operations, amplifying these complications. These complications also burden healthcare systems by increasing the need for additional operations, lengthening hospital stays, and raising readmission rates [4,34]. The cumulative economic burden—including surgical costs, hospitalization, and productivity losses—reaches several billion dollars annually in high-income countries. The unpredictability of adhesion-related events also hinders surgical planning and resource allocation, adding hidden costs. Given these consequences, preventing and effectively managing postoperative adhesions remain essential for improving surgical outcomes and reducing both healthcare and societal burdens [35].

Strategies to prevent postoperative adhesions can be broadly classified as physical or chemical barriers [1]. Physical barriers cover the surgical site to limit contact between injured and adjacent tissues, whereas chemical barriers interfere with the biological processes driving adhesion formation.

Most anti-adhesive agents in clinical use function as physical barriers [7]. They minimize adhesions at the application site but generally act only locally. Thus, they may not prevent adhesions that form distant from the surgical site, which can also cause substantial postoperative complications. In contrast, pathway-targeting chemical agents may offer broader and more sustained protection against both local and distant adhesions [1].

The peritoneal fibrinolytic system is a promising target. Under normal physiological conditions, the peritoneum contains minimal fibrin and fibrinogen, and early fibrinous adhesions (proto-adhesions) are rapidly degraded. This clearance reflects a fibrinolytic balance favoring t-PA over PAI-1 [36]. After peritoneal injury from surgical trauma, fibrin-rich exudate is secreted and fibrin deposition occurs within hours [37]. Concurrently, PAI-1 expression at the mRNA and protein levels increases disproportionately relative to t-PA, markedly reducing fibrinolytic activity [38]. This shift—characterized by excessive fibrin accumulation and impaired fibrin degradation—creates a biochemical environment conducive to adhesion formation.

Although NK-1RAs significantly increased t-PA activity, no significant changes were observed in t-PA or PAI-1 mRNA expression. This discrepancy likely reflects the time lag between mRNA transcription and protein activation, as well as post-transcriptional regulation of fibrinolytic pathways. The limited number of studies assessing mRNA expression and variation in tissue sampling times may also have contributed to this inconsistency.

Substance P acts centrally in this cascade. By activating neurokinin-1 receptors (NK-1R) on leukocytes and endothelial cells, substance P intensifies inflammation, promotes cytokine release, prolongs local inflammatory responses, and increases microvascular permeability [39]. These changes facilitate extravasation of fibrinogen into the peritoneal cavity. Moreover, substance P disrupts the t-PA–PAI-1 balance in a way that suppresses fibrinolysis, further promoting adhesion formation [40].

Blocking NK-1R with antagonists may therefore dampen early inflammation, reduce the intensity and duration of immune-cell infiltration, and indirectly restore fibrinolytic activity [40]. Consistent with this mechanism, animals treated with NK-1RAs had fewer macroscopic postoperative adhesions and significantly higher t-PA activity than controls, supporting the role of NK-1R blockade in enhancing fibrinolysis and preventing adhesion formation. In our trial sequential analysis, the cumulative Z-curve crossed the monitoring boundary, suggesting sufficient evidence for an anti-adhesive effect of NK-1RAs in preclinical models.

Because adhesiogenesis and wound healing share inflammatory and fibroproliferative pathways, interventions aimed at reducing adhesions may theoretically interfere with repair. However, in the limited available data, NK-1RA administration did not impair anastomotic healing. One study demonstrated comparable bursting pressures between NK-1RA and control groups [25], while another reported increased pressures in animals receiving valproic acid combined with an NK-1RA [29]. These findings suggest that NK-1RAs are unlikely to exert negative effects on anastomotic integrity and may have a neutral or potentially favorable influence on tissue healing.

This review had limitations. First, the meta-analysis showed substantial heterogeneity attributable to differences in animal species, surgical models, and experimental protocols. We addressed this via subgroup analyses by species and surgical type and sensitivity analyses for the primary outcome. We also conducted TSA to account for the small evidence base; the results supported an overall anti-adhesive effect of NK-1RAs despite between-study variability.

Second, with the exception of one study that used aprepitant [27], all included studies evaluated CJ-12,255, limiting generalizability across NK-1RAs. Further research using other NK-1RAs is warranted to confirm postoperative anti-adhesive effects across agents.

Third, the certainty of evidence, evaluated using the preclinical GRADE-based framework proposed by Hooijmans et al., was generally low to moderate because of methodological heterogeneity, small sample sizes, and unclear reporting of randomization or blinding. Nonetheless, the direction and magnitude of effects were consistent across independent studies, supporting a cautiously optimistic interpretation of NK-1RA efficacy in reducing postoperative adhesions.

Finally, because all included studies were preclinical, rigorously designed human clinical trials are needed before these findings can be applied to practice. Although preclinical results appear promising, the current evidence remains exploratory and should be interpreted as hypothesis-generating rather than directly translatable to clinical settings. Nonetheless, the present findings provide a scientific foundation for the rational design of clinical trials investigating NK-1RAs for adhesion prevention.

From a translational perspective, optimizing perioperative inflammation and fibrinolysis has direct implications for surgical outcomes. In laparoscopic and open abdominal procedures, intraoperative hemostatic techniques and local tissue responses can markedly influence postoperative adhesion formation. Recent clinical evidence shows that the choice of hemostatic approach affects tissue recovery and adhesion-related sequelae—for example, in laparoscopic endometrioma excision, where different hemostatic strategies variably impact ovarian reserve [41], and in laparoscopic liver surgery, where the method of bleeding control modulates both hemostasis and peritoneal healing [42]. These insights underscore the clinical relevance of maintaining a balanced hemostatic and fibrinolytic environment during surgery, consistent with the mechanistic rationale of NK-1R blockade observed in preclinical models.

Despite these limitations, the study has notable strengths—particularly rigorous methodology—and, to our knowledge, is the first systematic review and meta-analysis to evaluate the anti-adhesive effect of NK-1RAs in preventing postoperative adhesions.

## 5. Conclusions

NK-1RAs effectively reduced postoperative adhesions, as reflected by macroscopic adhesion scores and t-PA activity in preclinical models. These results are hypothesis-generating and provide a conceptual framework for future translational and clinical investigations exploring NK-1RAs as potential pharmacologic agents for adhesion prevention.

## Figures and Tables

**Table 1:** Characteristics of included studies.

First Author, Publication Year	Animal	Surgery	Group	Definition	
Reed, 2004 [24]	Male Wistar rats	Intraperitoneal ischemic buttons	Control 1 (n = 21)	Sterile saline; same schedule as Experiment 1, substituting saline for CJ-12,255	
			Experiment 1 (n = 21)	CJ-12,255; IP injection (2.5 mg/kg) twice daily for 2 days before surgery and for 7 days after surgery; IP lavage of 0.75 mg on the day of surgery	
			Control 2 (n = 6)	Sterile saline; same schedule as Experiment 2, substituting saline for CJ-12,255	
			Experiment 2	CJ-12,255; IP delivery (10 mg/kg) via an ALZET osmotic pump	
Cohen, 2007a [25]	Male Wistar rats	Intraperitoneal ischemic buttons	Control 1-1 (n = 6)	Sterile saline; same lavage schedule as Experiments 1-1/1-2, substituting saline for CJ-12,255	
			Experiment 1-1 (n = 6)	CJ-12,255; intraoperative IP lavage (5 mg/kg)	
			Experiment 1-2 (n = 6)	CJ-12,255; intraoperative IP lavage (25 mg/kg)	
			Control 2 (n = 4)	Sterile saline; same schedule as Experiment 2, substituting saline for CJ-12,255	
			Experiment 2 (n = 4)	CJ-12,255; IP injection (25 mg/kg) 1 h after surgery	
			Control 3 (n = 7)	Sterile saline; same schedule as Experiment 3, substituting saline for CJ-12,255	
			Experiment 3 (n = 7)	CJ-12,255; IP injection (25 mg/kg) 5 h after surgery	
			Control 4 (n = 4)	Sterile saline; same schedule as Experiment 4, substituting saline for CJ-12,255	
			Experiment 4 (n = 4)	CJ-12,255; IP injection (25 mg/kg) 12 h after surgery	
			Control 5 (n = 5)	Sterile saline; same schedule as Experiment 5, substituting saline for CJ-12,255	
			Experiment 5 (n = 5)	CJ-12,255; IP injection (25 mg/kg) 24 h after surgery	
Cohen, 2007b [26]	Male Wistar rats	Intraperitoneal ischemic buttons	Control 1 (n = 8)	Sterile water; same schedule as Experiment 1, substituting water for CJ-12,255; euthanized 1 day after surgery	
			Experiment 1 (n = 7)	CJ-12,255; intraoperative IP lavage (25 mg/kg); euthanized 1 day after surgery	
			Sham 1 (n = 7)	No surgery; euthanized 1 day after surgery	
			Control 2 (n = 8)	Sterile water; same schedule as Experiment 2, substituting water for CJ-12,255; euthanized 7 days after surgery	
			Experiment 2 (n = 7)	CJ-12,255; intraoperative IP lavage (25 mg/kg); euthanized 7 days after surgery	
Prushik, 2007 [30]	Male Wistar rats	Laparoscopic lysis of adhesions 7 days after intraperitoneal ischemic buttons	Control 1 (n = 12)	Sterile water; same schedule as Experiment 1, substituting water for CJ-12,255; euthanized 7 days after surgery	
			Experiment 1 (n = 11)	CJ-12,255; intraoperative IP lavage (25 mg/kg); euthanized 7 days after surgery	
			Self-control 1 (n = 23)	No injection; adhesions assessed during laparoscopy	
			Control 2 (n = 4)	Sterile water; same schedule as Experiment 2, substituting water for CJ-12,255; euthanized 1 day after surgery	
			Experiment 2 (n = 4)	CJ-12,255; intraoperative IP lavage (25 mg/kg); euthanized 1 day after surgery	
Lim, 2008 [27]	Male Wistar rats	Intraperitoneal ischemic buttons	Control 1 (n = 12)	Dimethyl sulfoxide (DMSO); same schedule as Experiment 1, substituting DMSO for aprepitant	
			Experiment 1 (n = 14)	Aprepitant; intraoperative IP lavage (50 mg/kg)	
			Control 2 (n = 7)	2% carboxymethylcellulose (CMC); oral gavage schedule as in Experiment 2 without aprepitant	
			Experiment 2 (n = 8)	Emend (aprepitant suspended in 2% CMC); single oral gavage (50 mg/kg) 3 h preoperatively	
			Control 3 (n = 14)	2% carboxymethylcellulose (CMC); oral gavage schedule as in Experiment 2 without aprepitant	
			Experiment 3 (n = 13)	Emend (aprepitant suspended in 2% CMC); oral gavage (50 mg/kg), five preoperative doses and then every 12 h over 60 h	
Lim, 2010 [28]	Male Wistar rats	Intraperitoneal ischemic buttons	Control 1 (n = 9)	Saline; same schedule as Experiment 1-1, substituting saline for CJ-12,255	
			Experiment 1-1 (n = 9)	CJ-12,255; IP lavage (25 mg/kg) before abdominal closure	
			Experiment 1-2 (n = 9)	1 × 1 cm HA/CMC pieces + saline; same procedure as Experiment 1-3, substituting saline for CJ-12,255	
			Experiment 1-3 (n = 9)	1 × 1 cm HA/CMC pieces + CJ-12,255; IP lavage (25 mg/kg) before abdominal closure	
			Control 2 ^a^ (n = 12)	Unilateral HA/CMC + saline; same schedule as Experiment 2a, substituting saline for CJ-12,255	
			Experiment 2 ^a^ (n = 12)	Unilateral HA/CMC + CJ-12,255; IP lavage (25 mg/kg) before abdominal closure	
Cassidy, 2014 [31]	Male wild-type mice/homozygous NK-1R−/− mice	Laparotomy with cecal cautery	Control 1 (n = 22; combined with Experiment 1)	Wild type; normal saline; same schedule as Experiment 1, substituting saline for CJ-12,255	
			Experiment 1	Wild type; CJ-12,255; intraoperative IP administration (25 mg/kg)	
			Control 2 ^a^ (n = 21; combined with Experiment 2)	Homozygous NK-1R−/− mice; same schedule as Experiment 2, substituting saline for CJ-12,255	
			Experiment 2 ^a^	Homozygous NK-1R−/− mice; CJ-12,255; intraoperative IP administration (25 mg/kg)	
Cassidy, 2015 [29]	Male Wistar rats	Intraperitoneal ischemic buttons	Control 1 (n = 9)	Normal saline; same schedule as Experiment 1-1, substituting saline for CJ-12,255	
			Experiment 1-1	Sodium valproate (VPA); IP administration (25 mg/kg) at the time of surgery	
			Experiment 1-2	CJ-12,255; IP administration (50 mg/kg) at the time of surgery	
			Experiment 1-3	Sodium valproate (VPA) + CJ-12,255; IP administration at the time of surgery: VPA (25 mg/kg) plus CJ-12,255 (50 mg/kg)	
			Control 2 (n = 6)	To assess normal wound healing; normal saline; same schedule as Experiment 2, substituting saline for CJ-12,255	
			Experiment 2 (n = 6)	To assess normal wound healing; sodium valproate (VPA) + CJ-12,255; IP administration at the time of surgery: VPA (25 mg/kg) plus CJ-12,255 (50 mg/kg)	
Kosaka, 2022 [32]	Female wild-type BALB/c mice; BALB/c NKT cell–deficient mice; BALB/c IFN-γ−/− mice	Laparotomy with cecal cautery	Control 1 (n = 7)	Laparotomy	Saline
			Experiment 1-1 (n = 7)	Laparotomy	SB225002; continuous IP infusion (800 μg) for 24 h after surgery using an ALZET 2001D osmotic pump
			Experiment 1-2 (n = 7)	Laparotomy	IP injection of CJ-12,255 (2.5 mg/kg) immediately after cecal cauterization
			Control 2 (n = 7)	Laparotomy + cauterization	Saline
			Experiment 2-1 (n = 7)	Laparotomy + cauterization	SB225002; continuous IP infusion (800 μg) for 24 h after surgery using an ALZET 2001D osmotic pump
			Experiment 2-2 (n = 7)	Laparotomy + cauterization	CJ-12,255; IP injection (2.5 mg/kg) immediately after cecal cauterization

IP, intraperitoneal. ^a^, Control 2 and Experiment 2 were conducted unilaterally with HA/CMC in the same rat.

**Table 2:** Definitions of the macroscopic adhesion score and other outcome.

First Author, Publication Year	Macroscopic Adhesion Score	Other Outcome Variables
Reed, 2004 [24]	Percentage of ischemic buttons with attached adhesions	Peritoneal tPA and PAI-1 mRNA expression; tPA-mediated fibrinolytic activity
Cohen, 2007a [25]	Percentage of ischemic buttons with attached adhesions	Peritoneal tPA and PAI-1 expression; fibrinolytic activity in peritoneal fluid; anastomotic integrity and healing (burst pressure)
Cohen, 2007b [26]	Percentage of ischemic buttons with attached adhesions	Matrix metalloproteinases (MMPs): total activity; MMP-2, -3, -7, -8, -9; TIMPs
Prushik, 2007 [30]	Percentage of ischemic buttons with attached adhesions	Fibrinolytic activity (tPA activity); tPA mRNA
Lim, 2008 [27]	Percentage of ischemic buttons with attached adhesions	tPA and PAI-1; tPA-mediated fibrinolytic activity
Lim, 2010 [28]	Percentage of ischemic buttons with attached adhesions	Peritoneal fibrinolytic activity
Cassidy, 2014 [31]	Adhesion scoring scale (0 = no adhesions; 1 = one thin, filmy adhesion; 2 = more than one thin adhesion; 3 = thick adhesion with a focal point; 4 = thick adhesion with planar attachment or more than one thick adhesion with a focal point; 5 = very thick, vascularized adhesion or more than one planar adhesion)	Fibrinolytic activity (tPA activity)
Cassidy, 2015 [29]	Percentage of ischemic buttons with attached adhesions	Anastomotic burst pressure
Kosaka, 2022 [32]	Adhesion scoring scale (0 = no adhesions; 1 = one thin, filmy adhesion; 2 = more than one thin adhesion; 3 = thick adhesion with a focal point; 4 = thick adhesion with planar attachment or more than one thick adhesion with a focal point; 5 = very thick, vascularized adhesion or more than one planar adhesion)	Cytokine/chemokine RNA expression; chemokine levels in abdominal lavage fluid

**Table 3:** Methodological quality assessment.

First Author and Publication Year	Random Allocation Stated	Husbandry Conditions Reported	Compliance with Animal Welfare Regulations	Potential Conflict of Interest	Peer Reviewed	Score
Reed 2004 [24]	1	1	1	0 (^a^)	1	4
Cohen 2007a [25]	1	1	1	0 (^a^)	1	4
Cohen 2007b [26]	1	1	1	0 (^a^)	1	4
Prushik 2007 [30]	1	1	1	0 (^a^)	1	4
Lim 2008 [27]	0	1	1	1	1	4
Lim 2010 [28]	1	1	1	0 (^a^)	1	4
Cassidy 2014 [31]	1	1	1	0 (^a^)	1	4
Cassidy 2015 [29]	0	1	1	0 (^a^)	1	3
Kosaka 2022 [32]	0	1	1	0 (^a^)	1	3

^a^ This item was downgraded because Pfizer, Inc. (Groton, CT, USA) supplied CJ-12,255, but the authors did not include a competing interests statement declaring no conflicts.

**Table 4:** Risk of bias assessed using SYRCLE’s tool.

First Author, Publication Year	Item 1	Item 2	Item 3	Item 4	Item 5	Item 6	Item 7	Item 8	Item 9	Item 10
**Reed, 2004** [24]	Unclear ^a^	Yes	Unclear ^b^	Yes	Unclear ^c^	Unclear ^d^	Yes	Yes	Yes	Unclear ^f^
**Cohen, 2007a** [25]	Unclear ^a^	Yes	Unclear ^b^	Yes	Unclear ^c^	Unclear ^d^	Yes	Yes	Yes	Unclear ^f^
**Cohen, 2007b** [26]	Unclear ^a^	Yes	Unclear ^b^	Yes	Unclear ^c^	Unclear ^d^	Yes	Yes	Yes	Unclear ^f^
**Prushik, 2007** [30]	Unclear ^a^	Yes	Unclear ^b^	Yes	Unclear ^c^	Unclear ^d^	Unclear ^e^	Yes	Yes	Unclear ^f^
**Lim, 2008** [27]	Unclear ^a^	Yes	Unclear ^b^	Yes	Unclear ^c^	Unclear ^d^	Unclear ^e^	Yes	Yes	Yes
**Lim, 2010** [28]	Unclear ^a^	Yes	Unclear ^b^	Yes	Unclear ^c^	Unclear ^d^	Yes	Yes	Yes	Unclear ^f^
**Cassidy, 2014** [31]	Unclear ^a^	Yes	Unclear ^b^	Yes	Unclear ^c^	Unclear ^d^	Yes	Yes	Yes	Unclear ^f^
**Cassidy, 2015** [29]	Unclear ^a^	Yes	Unclear ^b^	Yes	Unclear ^c^	Unclear ^d^	Unclear ^e^	Yes	Yes	Unclear ^f^
**Kosaka, 2022** [32]	Unclear ^a^	Yes	Unclear ^b^	Yes	Unclear ^c^	Unclear ^d^	Yes	Yes	Yes	Unclear ^f^
**Item**	**Description of domain**									
**Item 1**	Describe the methods used, if any, to generate the allocation sequence in sufficient detail to allow an assessment whether it should produce comparable groups.									
**Item 2**	Describe all the possible prognostic factors or animal characteristics, if any, that are compared in order to judge whether or not intervention and control groups were similar at the start of the experiment.									
**Item 3**	Describe the method used to conceal the allocation sequence in sufficient detail to determine whether intervention allocations could have been foreseen before or during enrolment.									
**Item 4**	Describe all measures used, if any, to house the animals randomly within the animal room.									
**Item 5**	Describe all measures used, if any, to blind trial caregivers and researchers from knowing which intervention each animal received. Provide any information relating to whether the intended blinding was effective.									
**Item 6**	Describe whether or not animals were selected at random for outcome assessment, and which methods to select the animals, if any, were used.									
**Item 7**	Describe all measures used, if any, to blind outcome assessors from knowing which intervention each animal received. Provide any information relating to whether the intended blinding was effective.									
**Item 8**	Describe the completeness of outcome data for each main outcome, including attrition and exclusions from the analysis. State whether attrition and exclusions were reported, the numbers in each intervention group (compared with total randomized animals), reasons for attrition or exclusions, and any re-inclusions in analyses for the review.									
**Item 9**	State how selective outcome reporting was examined and what was found.									
**Item 10**	State any important concerns about bias not covered by other domains in the tool.									

^a^: allocation sequence generation was not described. ^b^: allocation concealment was not described. ^c^: blinding of investigator was not described. ^d^: animal selection at random for outcome assessment was not described in the manuscript. ^e^: blinding of outcome assessor was not described. ^f^: This item was downgraded because Pfizer, Inc. (Groton, CT, USA) supplied CJ-12,255, but the authors did not include a competing interest statement declaring no conflicts.

**Table 5:** The GRADE evidence quality for each outcome assessed from pre-clinical studies.

	Study Limitations	Imprecision	Inconsistency	Indirectness	Publication Bias	GRADE
Macroscopic adhesion	Serious ^a^	Not serious	Serious ^c^	Not serious	Not serious	⨁⨁◯◯ Low
t-PA mRNA expression	Serious ^a^	Serious ^b^	Serious ^c^	Not serious	NA	⨁◯◯◯ Very low
PAI-1 mRNA expression	Serious ^a^	Serious ^b^	Serious ^c^	Not serious	NA	⨁◯◯◯ Very low
t-PA activity	Serious ^a^	Not serious	Serious ^c^	Not serious	NA	⨁⨁◯◯ Low
Anastomotic bursting pressure	Serious ^a^	Serious ^b^	Not serious	Not serious	NA	⨁⨁◯◯ Low

NA: not assessed, t-PA: tissue plasminogen activator, PAI-1: Plasmin Activator Inhibitor-1. ^a^ We downgraded study limitation as includes studies included studies exhibited a high risk of bias. ^b^ We downgraded imprecision by one level for serious imprecision due to very wide confidence intervals, including both substantial harms and benefits. ^c^ We downgraded the certainty of evidence by one level for inconsistency due to heterogeneity among studies, with an I^2^ statistic over 50% or P_chi2_ less than 0.05.

## Data Availability

All relevant data are within the manuscript and its Supporting Information Files.

## Supplementary Materials

The following supporting information can be downloaded at: https://www.mdpi.com/article/10.3390/medicina61111933/s1, Supporting Information File, PRISMA file [43].

## Abbreviations

The following abbreviations are used in this manuscript:

NK-1RA	Neurokinin-1 receptor antagonist
t-PA	tissue plasminogen activator
PAI-1	plasminogen activator inhibitor-1
SMD	standardized mean difference
MD	mean difference
CI	confidence interval
PrI	predictive interval
TSA	trial sequential analysis
RIS	required information size

## Appendix A

### Appendix A.1. Search Term

**Pubmed**	
#1	General Surgery[MeSH Terms]
#2	surg*[Title/Abstract]
#3	oper*[Title/Abstract]
#4	Surgical Procedures, Operative[MeSH Terms]
#5	surgery [Subheading]
#6	#1 OR #2 OR #3 OR #4 OR #5
#7	Tissue Adhesions[MeSH Terms]
#8	adhesi*[Title/Abstract]
#9	#7 OR #8
#10	#6 AND #9
#11	Neurokinin[Title/Abstract]
#12	NK-1[Title/Abstract]
#13	aprepitant[Title/Abstract]
#14	Emend*[Title/Abstract]
#15	Fosaprepitant*[Title/Abstract]
#16	Netupitant*[Title/Abstract]
#17	Akynzeo*[Title/Abstract]
#18	Casopitant*[Title/Abstract]
#19	Maropitant*[Title/Abstract]
#20	Rolapitant*[Title/Abstract]
#21	Tachykinin[Title/Abstract]
#22	#11 OR #12 OR #13 OR #14 OR #15 OR #16 OR #17 OR #18 OR #19 OR #20 OR #21
#23	#10 AND #22
**Embase**	
#1	‘general surgery’/exp OR ‘general surgery’
#2	surg*:ti,ab
#3	oper*:ti,ab
#4	‘surgery’/exp OR ‘surgery’
#5	#1 OR #2 OR #3 OR #4
#6	‘adhesion’/exp
#7	adhesi*:ti,ab
#8	‘adhesion barrier’/exp
#9	#6 OR #7 OR #8
#10	#5 AND #9
#11	‘tachykinin receptor antagonist’/exp
#12	neurokinin:ti,ab
#13	‘nk 1’:ti,ab
#14	aprepitant:ti,ab
#15	emend*:ti,ab
#16	fosaprepitant*:ti,ab
#17	netupitant*:ti,ab
#18	akynzeo*:ti,ab
#19	casopitant*:ti,ab
#20	maropitant*:ti,ab
#21	rolapitant*:ti,ab
#22	#11 OR #12 OR #13 OR #14 OR #15 OR #16 OR #17 OR #18 OR #19 OR #20 OR #21
#23	#10 AND #22
**Web of Science**	
#1	TS = Surgery
#2	TI = surg*
#3	AB = surg*
#4	TI = oper*
#5	AB = oper*
#6	#5 OR #4 OR #3 OR #2 OR #1
#7	TI = adhesi*
#8	AB = adhesi*
#9	TS = Tissue adhesion
#10	#9 OR #8 OR #7
#11	#10 AND #6
#12	TS = Neurokinin
#13	TI = NK-1
#14	AB = NK-1
#15	TI = NK1
#16	AB = NK1
#17	TI = aprepitant
#18	AB = aprepitant
#19	TI = Fosaprepitant
#20	AB = Fosaprepitant
#21	TI = Netupitant
#22	AB = Netupitant
#23	TI = Casopitant
#24	AB = Casopitant
#25	T I= Maropitant
#26	AB = Maropitant
#27	TI = Rolapitant
#28	AB = Rolapitant
#29	AB = Akynzeo
#30	AB = Emend
#31	#12 OR #13 OR #14 OR #15 OR #16 OR #17 OR #18 OR #19 OR #20 OR #21 OR #22 OR #23 OR #24 OR #25 OR #26 OR #27 OR #28 OR #29 OR #30
#32	#11 AND #31

## Appendix B

### Appendix B.1. Conference Proceedings Excluded

**No.**	**Reference No.**	**Brief Description**	**Reason for Exclusion**
1	Brady 2014 [44], Brady 2015 [45]	Two proceedings reporting identical content; [44] retained temporarily. Later matched to Cassidy et al., 2015 [29] with nearly identical authors and study population but different outcome; insufficient data in proceeding to extract effect estimates.	Duplicate content; insufficient data
2	Kosaka 2011 [46], Kosaka 2012 [32]	Two proceedings with identical content; [47] retained temporarily. Subsequently published as [32] by overlapping authors with similar population and outcomes.	Duplicate content; subsequent peer-reviewed publication
3	Cassidy 2012 [48]	Compared an intervention outside the scope of the review.	Irrelevant intervention

### Appendix B.2. Articles Excluded at the Full-Text Review Stage

**No.**	**Reference No.**	**Reason for Exclusion**
1	Reed 2008 [49]	Review article
2	Reed 2002 [50]	Did not report outcome of interest
3	Dehlin 2013 [51]	Did not report outcome of interest
4	Esposito 2013 [52]	Did not report outcome of interest
5	Gainsbury, 2011 [53]	Did not report outcome of interest
6	Azma 2014 [54]	Irrelevant intervention
